# Aminomethyl spectinomycins: a novel antibacterial chemotype for biothreat pathogens

**DOI:** 10.1038/s41429-019-0194-8

**Published:** 2019-06-04

**Authors:** Jennifer M. Scarff, Samanthi L. Waidyarachchi, Christopher J. Meyer, Douglas J. Lane, Weirui Chai, Margaret M. Lemmon, Jiuyu Liu, Michelle M. Butler, Terry L. Bowlin, Richard E. Lee, Rekha G. Panchal

**Affiliations:** 1 Molecular & Translational Sciences Division, United State Army Medical Research Institute of Infectious Diseases, Fort Detrick, Frederick, MD USA; 2 Department of Chemical Biology and Therapeutics, St. Jude Children’s Research Hospital, Memphis, TN USA; 3 Microbiotix, Worcester, MA USA

**Keywords:** Antibiotics, Pathogens

## Abstract

New antibiotics that are active against multi-drug-resistant strains and difficult-to-treat bacterial infections are needed. Synthetic modification of spectinomycin, a bacterial protein synthesis inhibitor, has been shown to increase antibacterial activity compared with spectinomycin. Aminomethyl spectinomycins are active against Gram-negative and Gram-positive bacterial pathogens. In this study, the ability of aminomethyl spectinomycins to treat biothreat pathogens is examined by MIC profiling, synergy testing, and in vivo efficacy experiments. Compound **1950** exhibited potent antibacterial activity against Gram-negative pathogens *Brucella* spp., *Burkholderia mallei*, and *Francisella tularensis*, but showed little to no growth inhibition against *Burkholderia pseudomallei*, *Bacillus anthracis*, and *Yersinia pestis*. Combination testing in checkerboard assays revealed that aminomethyl spectinomycin-antibiotic combinations had mainly an additive effect against the susceptible biodefense pathogens. The in vivo efficacy of compound **1950** was also demonstrated in mice infected with *B. mallei* (FMH) or *F. tularensis* (SchuS4). These results suggest that aminomethyl spectinomycins are promising new candidates for development of therapeutics against biodefense bacterial agents.

## Introduction

The rate of emergence of antibiotic-resistant pathogens is outpacing the development of novel antimicrobial agents [1]. One approach to develop new antibiotics is to synthetically modify existing under-utilized antibiotics, such as spectinomycin. Spectinomycin is an aminocyclitol antibiotic that, although structurally similar to the aminoglycoside class, binds to a unique site in RNA helix 34 of the 30S ribosomal subunit, blocks translocation, and halts protein translation [2–4]. Spectinomycin was successfully used in treatments of *Neisseria gonorrhoeae* until resistance arose, and it was withdrawn from frontline use in the United States [5]. Nevertheless, spectinomycin demonstrates a high safety margin and has a low-molecular-weight which makes it an ideal antibiotic for synthetic modification.

**Fig. 1:**
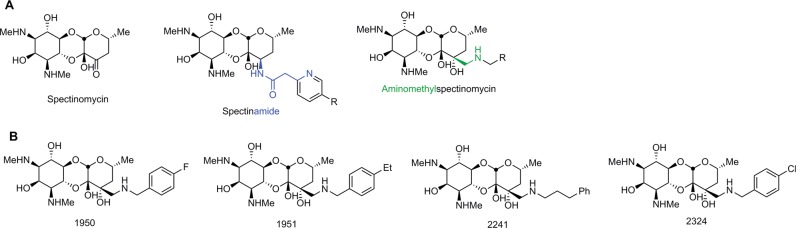
**a** General structures of modified spectinomycin analogs; **b** Structures of the aminomethyl spectinomycins examined in this study. Note: AmSPCs **1950**, **1951**, and **2241** exist as the trihydrochloride salts; **2324** exists as the trihydrobromide salt. See the Materials and methods section for details

Previous work has demonstrated that spectinomycins with amide modifications on the 3′ position, also called “spectinamides” (Fig. 1a), have increased activity against multi-drug-resistant strains of *Mycobacterium tuberculosis*. Critical to this series is the addition of a pyridyl side-chain motif that enables the spectinamide to avoid the Rv1258c efflux pump [6, 7]. Recently, novel *N*-benzyl aminomethyl spectinomycins (amSPCs) demonstrated broad spectrum in vitro activity against Gram-positive and Gram-negative bacterial pathogens. The amSPCs were active against multi-drug-resistant strains of *Streptococcus pneumoniae* and *N. gonorrhoeae* and were also able to protect mice from a lethal pulmonary challenge with *S. pneumoniae* [8, 9]. Although the new spectinomycin analogs had improved activity, they were not active against all drug-resistant strains of *N. gonorrhoeae*. This clearly demonstrates that modification of spectinomycin can result in more potent broad-spectrum antibiotics, and that both the amSPCs and spectinamides are efficacious in treating pulmonary infections in animal models.

There are bacteria of importance to biodefense with natural and acquired antibiotic resistance. *Burkholderia pseudomallei*, in particular, is resistant to penicillins, cephalosporins, quinolones, rifampin, and aminoglycosides [10–12]. While *Burkholderia mallei* is resistant to fewer antibiotics than *B. pseudomallei*, it is also naturally resistant to multiple classes of antibiotics [10, 13, 14]. Treatment of *Francisella tularensis* is limited to the use of three antibiotic classes: fluoroquinolones, tetracyclines, and aminoglycosides. Although *F. tularensis* strains resistant to these antibiotics have not yet been reported, treatment failures in up to 25% of cases have occurred [15–17]. Facing the threat that these dangerous pathogens pose, new antibiotics that are active against these critical agents are needed.

Here, we report the testing of amSPCs against biothreat bacterial pathogens *B. mallei*, *B. pseudomallei*, *F. tularensis*, *Brucella* spp., *Bacillus anthracis*, and *Yersinia pestis*. Lead amSPCs possess improved activity against several of these pathogens compared with spectinomycin. In addition, treatment with an amSPC led to an increased survival rate of mice infected with *B. mallei* or *F. tularensis*.

## Materials and methods

### Synthesis of the amSPCs

Compounds **1950**, **1951**, **2241**, and **2324** were synthesized according to the procedure reported previously [8]. The purity and identity of the compounds was determined using reverse phase Ultra-Performance Liquid Chromatography (UPLC) analysis with Evaporating Light Scattering Detector MS (ELSD/MS) detection and by ^1^H and ^13^C NMR spectroscopy. All compounds were confirmed to be >95% pure before being submitted for biological testing. Elemental analysis of select compounds was performed by Atlantic Microlabs to confirm salt composition.

3′-(*R*)-3′-(*N*-(4-fluorobenzyl)aminomethyl)dihydrospectinomycin trihydrochloride (**1950**)

^1^H NMR (D_2_O, 400 MHz): *δ* 7.34 (dd, *J* = 3.6, 10.3 Hz, 2H), 7.12–7.07 (m, 2H), 4.51 (s, 1H), 4.48 (s, 1H), 3.98 (t, *J* = 10.5 Hz, 1H), 3.90 (d, *J* = 13.4 Hz, 1H), 3.86–3.76 (m, 2H), 3.73–3.73 (m, 2H), 2.99 (d, *J* = 13.5 Hz, 1H), 2.88–2.79 (m, 3H), 2.57 (s, 3H), 2.42 (s, 3H), 1.75–1.60 (m, 2H), 1.13 (d, *J* = 6.0 Hz, 3H). ^13^C NMR (126 MHz, D_2_O): *δ* 163.20(d), 132.25, 132.18, 125.96, 116.24, 116.06, 93.17, 92.31, 72.16, 69.59, 67.31, 65.87, 65.28, 61.45, 59.46, 58.43, 50.74, 48.88, 40.00, 30.55, 30.29, 19.84. High-resolution electrospray ionization mass spectrometry (HRMS-ESI) calculated for C_22_H_34_FN_3_O_7_ [M+H]^+^ 472.2459, found 472.2459. Elemental analysis theoretical composition for empirical formula C_22_H_34_FN_3_O_7_∙3 HCl∙2.5 H_2_O: C, 42.46; H, 6.74; N, 6.75; Cl, 17.09; F, 3.05. Found formula for C_22_H_34_FN_3_O_7_∙3 HCl∙2.5 H_2_O: C, 42.21; H, 6.86; N, 6.52; Cl, 16.73; F, 2.85.

3′-(*R*)-3′-(*N*-(4-ethylbenzyl)aminomethyl)dihydrospectinomycin trihydrochloride (**1951**)

^1^H NMR (D_2_O, 400 MHz): *δ* 7.44 (d, *J* = 7.6 Hz, 2H), 7.40 (d, *J* = 7.6 Hz, 2H), 4.74 (s, 1H), 4.33–4.20 (m, 3H), 4.01 (t, *J* = 10 Hz, 1H), 3.94 (t, *J* = 10 Hz, 1H), 3.69 (m, 1H), 3.50 (m, 1H), 3.38 (ABq, *J* = 14.0 Hz, 1H), 3.17 (ABq, *J* = 14.0 Hz, 1H), 2.81 (s, 3H), 2.76 (s, 3H), 2.68 (m, 2H), 1.82 (m, 2H), 1.22 (m, 8H). ^13^C NMR (126 MHz, D_2_O): *δ* 146.68, 130.10, 128.77, 127.39, 93.20, 92.35, 72.12, 69.80, 67.27, 66.01, 65.48, 61.58, 59.60, 58.54, 51.11, 48.76, 40.06, 30.54, 30.35, 27.97, 19.84, 14.84. HRMS-ESI calculated for C_24_H_39_N_3_O_7_ [M+H]^+^ 482.2788, found 482.2873.

3′-(*R*)-3′-(N-(3-phenylpropyl)aminomethyl)dihydrospectinomycin trihydrochloride (**2241**)

^1^H NMR (D_2_O, 400 MHz): *δ* 7.40 (m, 2H), 7.32 (m, 3H), 4.89 (s, 1H), 4.67 (t, *J* = 4 Hz, 1H), 4.15 (t, *J* = 12.0 Hz, 1H), 4.01 (t, *J* *=* 8.0 Hz, 1H), 3.90 (t, *J* = 8.0 Hz, 1H), 3.84 (m, 1H), 3.43 (ABq, *J* = 14.0 Hz, 1H), 3.22 (br, 2H), 3.10 (m, 3H), 2.77 (s, 3H), 2.74 (m, 2H), 2.63 (s, 3H), 2.05 (m, 2H), 1.88 (d, *J* = 13.2 Hz, 1H), 1.78 (d, *J* = 13.2 Hz, 1H), 12.5 (d, *J* *=* 6.0 Hz, 3H). ^13^C NMR (126 MHz, D_2_O): *δ* 128.80, 128.53, 128.47, 126.50, 96.35, 93.23, 70.02, 67.32, 66.19, 65.72, 61.69, 59.77, 58.64, 50.22, 48.83, 48.15, 42.79, 40.04, 31.84, 30.61, 30.46, 26.59, 23.19, 19.88. HRMS-ESI calculated for C_24_H_39_N_3_O_7_ [M+H]^+^ 482.2866, found 482.2860. Elemental analysis theoretical composition for the empirical formula C_24_H_39_N_3_O_7_∙3 HCl∙2.3 H_2_O: C, 45.58; H, 7.43; N, 6.64; Cl, 16.82. Found formula for C_22_H_34_FN_3_O_7_∙3 HCl∙2.3 H_2_O: C, 45.36; H, 6.96; N, 6.54; Cl, 16.66.

3′-(*R*)-3′-(*N*-(4-chlorobenzyl)aminomethyl)dihydrospectinomycin trihydrobromide (**2324**)

^1^H NMR (D_2_O, 400 MHz): *δ* 7.48–7.37 (m, 4H), 4.30–4.16 (m, 3H), 3.97 (t, *J* = 9.9 Hz, 1H), 3.88 (t, *J* = 10.0 Hz, 1H), 3.73–3.62 (m,1H), 3.50–3.45 (m, 1H), 3.33 (d, *J* = 13.6 Hz, 1H), 3.26 (s, 2H), 3.21–3.12 (m, 2H), 2.75 (s, 6H), 1.83–1.67 (m, 2H), 1.15 (d, *J* = 6.0 Hz, 3H). ^13^C NMR (126 MHz, D_2_O): *δ* 135.28, 131.57, 129.34, 128.53, 93.16, 92.32, 72.18, 69.56, 67.33, 65.86, 65.28, 61.43, 59.45, 58.43, 50.76, 49.03, 40.02, 30.60, 30.37, 19.87. HRMS-ESI calculated for C_22_H_34_ClN_3_O_7_ [M+H]^+^ 488.2164, found 488.2169.

### MIC assay

The MICs were determined as recommended by CLSI in 96-well plates with compound at an initial concentration of 200 µg ml^−1^ and serially diluted twofold. Approximately 5 × 10^4^ CFU of bacteria in CAMHB (or CAMHB supplemented with 2% IsoVitalex for *F. tularensis*) were added to each well and plates were incubated at 37°C for 24–48 h. The MIC was determined as the lowest concentration of the compound that prevented growth of the bacterial strain. The MIC_50_ and MIC_90_ values were determined as the concentration of the amSPCs at which growth was inhibited in 50 and 90%, respectively, of the strains that were tested.

The MICs of spectinomycin and compound **1950** were also tested in combination with other antibiotics or polymyxin B nonapeptide (PMBN). For the dual antibiotic studies, a checkerboard serial dilution of antibiotics was completed in 96-well plates so that, when possible, the MIC of an individual antibiotic was in the middle of the dilutions (i.e., there were at least two dilutions above and below the MIC in the checkerboard). The bacteria were applied to the plates and incubated as described above. The fractional inhibitory concentration (FIC) index was calculated with the following formula: FIC = (A/MIC_A_) + (B/MIC_B_), where MIC_A_ and MIC_B_ are the MIC of each drug individually and A and B are the MICs of each antibiotic in combination. Effects were determined to be synergistic (FIC ≤ 0.5), additive (0.5 < FIC ≤ 1.0) indifferent (1.0 < FIC ≤ 4.0), or antagonistic (FIC > 4.0).

For the PMBN combination studies, a checkerboard serial dilution of spectinomycin or compound **1950** (initial concentration 64 µg ml^−1^) and PMBN (initial concentration 32 µg ml^−1^) were completed in a 96-well plate. *F. tularensis* LVS or *B. pseudomallei* strain Bp82 were applied to the plate as described above and incubated at 37°C with 5% CO_2_ for 40–48 h. The MIC of spectinomycin or **1950** was recorded for each concentration of PMBN.

### Time-kill assay

An overnight culture of *B. mallei* strain FMH in CAMHB was diluted to an OD_600_ of 0.01 and incubated, with shaking, at 37 °C for 6 h to allow the culture to reach the mid-exponential phase of growth. The culture was then diluted to a concentration of 5 × 10^5^ CFU ml^−1^. Compound **1950**, spectinomycin, or an equal volume of DMSO/diluent was added such that the final concentration of compound/antibiotic was 50 µg ml^−1^ and DMSO was 0.5%. The samples were incubated at 37°C with aeration. A sample was removed from the culture at 0, 1.25, 2.5, 4, 6, and 24 h for serial dilution and enumeration of the bacteria on sheep blood agar (SBA) plates. Significant differences between the groups at each time point were determined by multiple *t* tests with the Holm-Šídák method to correct for multiple comparisons in GraphPad Prism 7.

### Mouse infection and colonization

Research was conducted under an IACUC approved protocol in compliance with the Animal Welfare Act, PHS Policy, and other Federal statutes and regulations relating to animals and experiments involving animals. The facility where this research was conducted is accredited by the Association for Assessment and Accreditation of Laboratory Animal Care, International and adheres to principles stated in the Guide for the Care and Use of Laboratory Animals, National Research Council, 2011. Six- to eight-week-old BALB/c mice (Charles River Laboratories/NCI, Frederick, MD) were used for all experiments.

Mice received an intranasal (i.n.) instillation of 3 × 10^4^ CFU *B. mallei* strain FMH in 20 µl. Treatment with amSPC **1950** (25 or 50 mg kg^−1^) or saline was initiated at 1 h post-infection. Treatments were administered twice daily (BID) via s.c. injection in 200 µl for 14 days. Mice were closely monitored for morbidity and mortality. On day 40, five mice were removed from the **1950**-treated groups (25 and 50 mg kg^−1^) for necropsy and were marked as censored subjects for the survival curve. Mice that survived until day 60 were euthanized and necropsy was completed. The lungs, livers, and spleens of mice were removed, homogenized, and CFU g^−1^ of tissue was determined.

To determine if a delay in initiation of treatment had an effect on survival of mice post-infection, mice received an i.n. inoculum of 2 × 10^4^ CFU *B. mallei* strain FMH in 20 µl. Treatment with **1950** (50 mg kg^−1^, BID), administered s.c. in 200 µl, was initiated at 1, 6, 24, or 48 h post infection and continued for 14 days. Control mice were administered saline or spectinomycin (50 mg kg^−1^, BID) starting at 1 h post infection. Mice were closely monitored for morbidity and mortality for 42 days.

Mice were infected i.n. with 1 × 10^3^ CFU *F. tularensis* strain SchuS4 in 20 µl. Treatment with compound **1950** (50 or 75 mg kg^−1^) or spectinomycin (50 mg kg^−1^) in 200 µl was initiated at 1 h post-infection and continued BID for 14 days. Mice were closely monitored for morbidity and mortality for 55 days. In a second experiment, mice were infected with 900 CFU *F. tularensis* SchuS4 and s.c. administration of saline, spectinomycin (50 mg kg^−1^), or **1950** (50 mg kg^−1^) in 200 µl was initiated at 1 h post-infection and continued three times daily (TID) for 14 days. Mice were closely monitored for morbidity and mortality for 37 days.

Significant differences in survival between groups were determined by log-rank (Mantel–Cox) test in GraphPad Prism 7. Significant differences in bacterial load were determined by Mann–Whitney test in GraphPad Prism 7.

## Results

### AmSPCs are active against *B. mallei*, *Brucella* spp., and *F. tularensis*

The MICs were determined for four amSPCs (structures shown in Fig. 1b) against panels of representative isolates of *B. mallei*, *Brucella* spp., *F. tularensis*, *B. anthracis*, and *B. pseudomallei*. The four amSPCs investigated had lower MIC_50_ and MIC_90_ values than those observed for spectinomycin for all species tested, except *B. pseudomallei* (Table 1). The *Brucella* spp. (*Brucella suis*, *Brucella abortus*, and *Brucella melitensis*) were most susceptible to the amSPCs; the 29 strains tested had an MIC range of 0.4–12.5 µg ml^−1^ (Table 1; Table S1). An increase in potency of the amSPCs over spectinomycin was also observed in the 29 strains of *B. mallei*; the MICs ranged from 1.56 to 50 µg ml^−1^ (Table 1; Table S2). Twenty-eight *B. mallei* strains had MICs that were ≤12.5 µg ml^−1^ and only one strain of *B. mallei* had an MIC value of 50 µg ml^−1^ for all four amSPC compounds that were tested (Table S2). *F. tularensis* showed a broad range of sensitivity to the amSPCs; the MICs ranged from 1.56 to 200 µg ml^−1^ across 26 strains (Table 1; Table S3). *B. anthracis* was less susceptible to the amSPCs with an MIC range of 3.13–100 µg ml^−1^ (Table 1; Table S4). For *B. anthracis*, the MIC_50_ values for the compounds were 12.5 or 25 µg ml^−1^, compared with the MIC_50_ for spectinomycin, 64 µg ml^−1^. *Y. pestis* strain CO92 and *B. pseudomallei* strain K96243 were less sensitive to the amSPCs, with MICs ≥25 µg ml^−1^ or ≥200 µg ml^−1^, respectively (Table 1).

**Table 1:** Antibacterial activity of amSPC compounds

		MIC (µg ml^-1^)				
Pathogen		1950	1951	2241	2324	SPT^a^
*B. mallei*	MIC Range^b^	3.13–50	3.13–50	3.13–50	1.56–50	8–16
	MIC_50_	6.3	6.3	6.3	6.3	8
	MIC_90_	12.5	12.5	12.5	12.5	16
*Brucella* spp.^c^	MIC Range^b^	0.4–12.5	0.4–12.5	0.4–12.5	≤0.2–6.3	1–32
	MIC_50_	1.56	3.13	1.56	1.56	4
	MIC_90_	3.13	6.25	3.13	6.25	8
*F. tularensis*	MIC Range^b^	1.56–200	3.13–200	3.13–200	1.56–200	1->64
	MIC_50_	12.5	6.25	12.5	6.25	64
	MIC_90_	100	50	50	50	>64
*B. anthracis*	MIC Range^b^	12.5–100	6.25–50	3.13–25	12.5–50	16->64
	MIC_50_	25	25	12.5	25	64
	MIC_90_	50	50	12.5	25	64
*Y. pestis* CO92	MIC	50	50	25	50	32
*B. pseudomallei* ^d^	MIC	>200	>200	>200	>200	>64

^a^SPT: spectinomycin

^b^The range of MIC values for 29 strains of *B. mallei*, 29 strains of *Brucella* spp., 26 strains of *F. tularensis*, and 30 strains of *B. anthracis*

^c^Brucella species tested: *B. abortus*, *B. suis*, and *B. melitensis*

^d^MIC was the same for all 10 *B. pseudomallei* strains tested

### Activity of amSPCs in combination with other antibiotics

Prior in vitro studies have shown that spectinomycins and spectinamides exhibit synergistic activity with different classes of antibiotics [18, 19]. To investigate possible synergistic effects, spectinomycin and amSPC **1950** were tested in combination with at least three antibiotics against each of the six biodefense pathogens, and the FIC index was determined for each combination (Table 2). Ten combinations for spectinomycin and 12 combinations for Lee **1950** had additive effects. Eight combinations for spectinomycin and nine combinations with Lee **1950** had indifferent combinations. Only three combinations for spectinomycin possessed FIC indices that would classify the combination as synergistic: spectinomycin with doxycycline or trimethoprim against *B. mallei* and with ciprofloxacin against *Y. pestis*. None of the combinations with **1950** had an FIC that would indicate synergy. There was one antagonistic combination observed with **1950** and streptomycin against *B. anthracis*.

**Table 2:** FIC indices for spectinomycin and compound **1950** in combination with other antibiotics

Bacterial strain	*B. pseudomallei* K96243		*B. mallei* FMH		*B. anthracis* Ames		*B. suis*		*F. tularensis* SchuS4		*Y. pestis* CO92	
MIC used for FIC calculation^a^	0.5 X MIC	0.25 X MIC	0.5 X MIC	0.25 X MIC	0.5 X MIC	0.25 X MIC	0.5 X MIC	0.25 X MIC	0.5 X MIC	0.25 X MIC	0.5 X MIC	0.25 X MIC
*Spectinomycin FIC*												
Doxycycline	1.5	1.25	0.62	0.5	0.75	0.75	0.74	1.25	0.625	0.75	0.75	0.75
Ciprofloxacin	–	–	–	–	1.5	1.25	–	–	1.5	2.25	0.74	0.49
Streptomycin	–	–	–	–	2.5	2.25	1.5	1.25	2.5	2.25	1	1.25
Chloramphenicol	–	–	–	–	–	–	–	–	0.75	0.75	1	1.25
Clarithromycin	–	–	–	–	1	0.75	–	–	–	–	–	–
Trimethoprim	1.5	1.25	0.50	0.375	–	–	1.5	1.25	–	–	n.d.^b^	n.d.^b^
Ceftazidime	1.5	0.75	1	0.75	–	–	–	–	–	–	–	–
*1950 FIC*												
Doxycycline	2.5	1.25	0.62	0.75	0.75	0.75	0.74	1.25	0.75	0.75	0.625	0.75
Ciprofloxacin	–	–	–	–	2.58	2.33	–	–	1.5	1.25	1	1.25
Streptomycin	–	–	–	–	4.5	4.25	2.5	1.25	2.5	2.25	1.5	1.25
Chloramphenicol	–	–	–	–	–	–	–	–	1.0	0.75	0.75	0.75
Clarithromycin	–	–	–	–	1	0.75	–	–	–	–	–	–
Trimethoprim	1.5	1.25	0.56	1.25	–	–	1.5	1.25	–	–	0.75	0.75
Ceftazidime	2.5	2.25	0.75	1.25	–	–	–	–	–	–	–	–

^a^MIC of Spectinomycin or compound **1950** used for FIC calculation

^b^Could not be calculated

We also tested polymyxin B nonapeptide (PMBN) in combination with spectinomycin and **1950** against *F. tularensis* live vaccine strain (LVS) and *B. pseudomallei* Bp82 (Table 3). PMBN binds to the bacterial cell membrane and can lower the MIC values of antibiotics [20]. PMBN was unable to reduce the MIC for spectinomycin or **1950** against *B. pseudomallei* Bp82; the MIC remained >64 µg ml^−1^ at a concentration of 32 µg ml^−1^ of PMBN. *F. tularensis* LVS was susceptible to PMBN at a concentration of 32 µg ml^−1^. When spectinomycin was combined with either 16 or 8 µg ml^−1^ PMBN, the MIC was reduced from 16 (spectinomycin alone) to 8 µg ml^−1^. The addition of 16 µg ml^−1^ PMBN reduced the MIC of **1950** from 4 to 1 µg ml^−1^. These data indicate that there are possible combination therapies that can improve the efficacy of amSPCs against biodefense pathogens.

**Table 3:** MICs of spectinomycin and compound **1950** in combination with PMBN

Bacteria Strain	MIC with PMBN (µg ml^−1^)					PMBN^a^
	0	4	8	16	32	
*Spectinomycin*						
*B. pseudomallei* Bp82	>64	>64	>64	>64	>64	>32
*F. tularensis* LVS	16	16	8	8	0.125	32
*Compound 1950*						
*B. pseudomallei* Bp82	>64	>64	>64	>64	>64	>32
*F. tularensis* LVS	4	4	4	1	0.125	32

^a^MIC (µg ml^-1^) of PMBN without antibiotic

### AmSPC 1950 is bactericidal against *B. mallei*

**Fig. 2:**
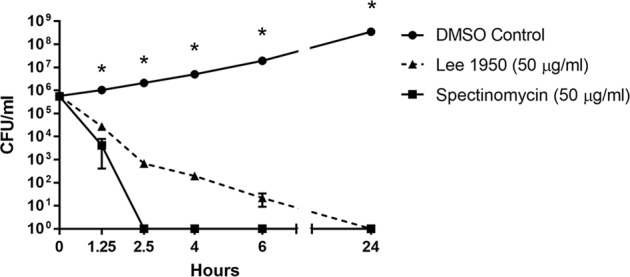
Bactericidal activity of **1950** against *B. mallei*. *B. mallei* FMH was cultured in the presence of 50 µg ml^−1^ compound **1950**, spectinomycin, or an equal dilution of DMSO (control). Enumeration of bacteria was completed at 0, 1.25, 2.5, 4, 6, and 24 h after initiation of the assay. *Significantly different from the untreated control group (*P* < 0.0004) as determined by multiple *t* tests with the Holm–Šídák method

The ability of amSPC **1950** and spectinomycin to kill *B. mallei* in vitro was assayed over a 24 h time frame (Fig. 2). By t_1.25_, there was a greater than 10-fold decrease and a greater than 100-fold decrease in the amount of viable bacteria in the **1950**-treated and spectinomycin-treated groups, respectively, compared with that of the DMSO control group. There was a decline in the number of bacteria present at each time point in the **1950**-treated group until no bacteria were detected at t_24_. Bacteria were not detected in the spectinomycin-control group from t_2.5_ through the end of the assay. There were statistically fewer bacteria in both the **1950**-treated group and the spectinomycin-treated group from t_1.25_ to t_24_ (*P* < 0.0004). These data indicate that amSPC **1950** is bactericidal against *B. mallei*.

### AmSPC 1950 improves *B. mallei* infection outcome

**Fig. 3:**
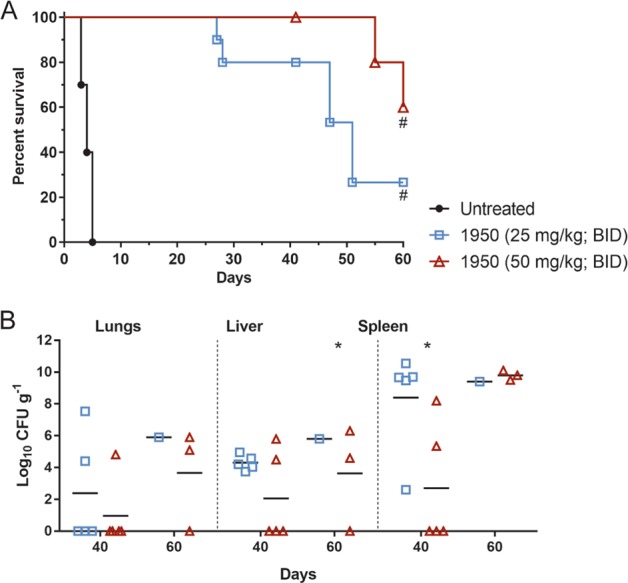
Effect of compound **1950** on *B. mallei* infection. **a** Survival of BALB/c mice (n = 10) infected i.n. with 3 x 10^4^ CFU *B. mallei* strain FMH. Treatment with saline (untreated) or **1950** (25 or 50 mg kg^−1^, BID) administered s.c. was initiated at 1 h post-infection and continued for 14 days. Mice were monitored for morbidity and mortality for 60 days. ^#^Significantly different from untreated control mice (*P* < 0.0001). **b** Bacterial load in the lungs, livers, and spleens of mice on day 40 (*n* = 5) or day 60 (*n* = 3, 50 mg kg^−1^; *n* = 1; 25 mg kg^−1^) post infection. Each symbol represents the CFU g^−1^ tissue for a mouse and the bars represent the mean. The five mice removed for determination of bacterial load on day 40 were censored on the survival curve on that date. *Significantly different from **1950** (50 mg kg^−1^) spleen, day 60 (*P* ≤ 0.0357) as determined by Mann–Whitney test

In addition to its in vitro potency, amSPC **1950** was selected for murine studies due to its improved half-life compared with spectinomycin and previously demonstrated efficacy against other pathogens [8, 9]. We determined whether **1950** could protect BALB/c mice from an i.n. infection with 3 × 10^4^ CFU *B. mallei* strain FMH (10 x LD_50_). Treatment with **1950** (25 or 50 mg kg^−1^, BID, s.c.) was initiated at 1 h post-inoculation and continued for 14 days. The mice that received no treatment succumbed to infection by day 5 post-infection. Treatment with **1950** resulted in an increased survival of infected mice; 10/10 mice that received **1950** (50 mg kg^−1^, BID) and 8/10 of mice that received **1950** (25 mg kg^−1^, BID) survived until day 40 post-infection (Fig. 3a). On day 40, five mice from each treatment group were euthanized to determine the bacterial load within different tissues and the remaining mice were monitored until day 60. Three mice that received **1950** (50 mg kg^−1^, BID) and one mouse that received **1950** (25 mg kg^−1^, BID) survived until day 60 post-challenge. The survival of mice that received either dose of **1950** was significantly different from the survival of the untreated control mice (*P* < 0.0001), but there was no difference in survival between the two treatment groups.

The bacterial load in the lungs, livers, and spleens were determined in mice from the **1950** treatment groups (25 and 50 mg kg^−1^, BID) on day 40. Only two mice that received **1950** (50 mg kg^−1^, BID) had detectable levels of bacteria in their lungs, livers, and spleens (Fig. 3b). In contrast, all five mice that received **1950** (25 mg kg^−1^, BID) had bacteria in their livers and spleens while two of these mice also had bacteria in their lungs. The decreased bacterial load in the mice that received **1950** (50 mg kg^−1^, BID) is indicative of a dose-dependent response. Furthermore, the bacterial load in the spleens of mice that received **1950** (50 mg kg^−1^, BID) was significantly different from those that received **1950** (25 mg kg^−1^, BID) (*P* = 0.0317). The tissue burden in the mice that survived until day 60 post-infection in the **1950** (50 mg kg^−1^, BID) group (*n* = 3) and the **1950** (25 mg kg^−1^, BID) group (*n* = 1) was also determined (Fig. 3b). Of the three mice that survived the infection after treatment with **1950** (50 mg kg^−1^, BID), two mice had bacteria in their lungs, livers, and spleens and one mouse only had bacteria in its spleen on day 60. In the one mouse that survived in the **1950** (25 mg kg^−1^, BID) group, bacteria were present in its lungs, liver, and spleen. There was a trend that the bacterial load in the mice increased from day 40 post infection to day 60 post infection for the **1950** (25 mg kg^−1^, BID) and **1950** (50 mg kg^−1^, BID) treatment groups, although the only significant difference was in the spleen between the **1950** (50 mg kg^−1^, BID) day 40 and **1950** (50 mg kg^−1^, BID) day 60 groups (*P* = 0.0357). This suggests that there is an expansion of the bacteria that survived the 14-day treatment regimen.

**Fig. 4:**
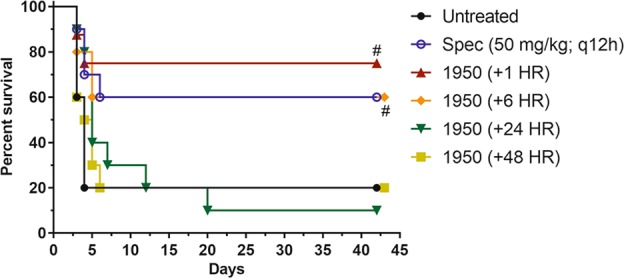
Survival of BALB/c mice infected i.n. with 2 × 10^4^ CFU *B. mallei* FMH. Treatment with **1950** (50 mg kg^−1^, BID) was initiated at 1, 6, 24, or 48 h post-infection and was administered s.c. for 14 days. Control mice were treated with saline (untreated) or spectinomycin (50 mg kg^−1^, BID) starting at 1 h post-infection. **1950** (1 h) group: *n* = 8; all other groups: *n* = 10. ^#^Significantly different from untreated control group (*P* < 0.05)

We also tested whether the time of treatment initiation post-infection altered the outcome in a *B. mallei* mouse infection (Fig. 4). Mice were infected i.n. with 2 x 10^4^ CFU *B. mallei* strain FMH and s.c. treatment with **1950** (50 mg kg^−1^) was initiated at 1, 6, 24, or 48 h post infection and continued for 14 days (BID). Untreated control mice had 2/10 mice survive until day 42 post-infection. Initiation of treatment at 24 or 48 h post-infection resulted in survival that was similar to the untreated control mice, 1/10 and 2/10, respectively. In comparison, treatment initiation at 1 or 6 h post-infection resulted in survival of 6/8 or 6/10 mice, respectively. The mice that received **1950** at 1 or 6 h post-infection had a significant increase in survival compared with the untreated control mice (*P* < 0.05). Initiation of spectinomycin (50 mg kg^−1^, BID) at 1 h post-infection resulted in the survival of 6/10 mice, which was similar to the survival observed after **1950** treatment. These data indicate that 50 mg kg^−1^ is an effective dose of **1950** when administered up to 6 h post-infection.

### AmSPC 1950 increases mice survival after *F. tularensis* infection

**Fig. 5:**
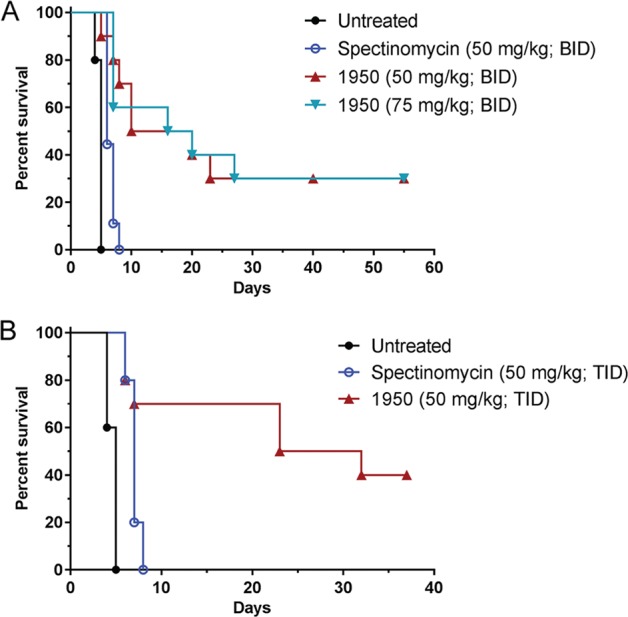
Effect of compound **1950** on *F. tularensis* infection. **a** Survival of mice (*n* = 10) infected i.n. with 1 × 10^3^ CFU *F. tularensis* strain SchuS4 (10 x LD_50_). Treatment with saline (untreated), spectinomycin (50 mg kg^−1^, BID), or **1950** (50 or 75 mg kg^−1^, BID) administered s.c. started at 1 h post-infection and continued for 14 days. Mice were monitored for morbidity and mortality for 55 days. ^#^Significantly different from untreated control (*P* < 0.0001) and spectinomycin control (*P* ≤ 0.0013). **b** Survival of mice infected i.n. with 900 CFU *F. tularensis* strain SchuS4 (10 x LD_50_). Treatment with saline (untreated), spectinomycin (50 mg kg^−1^, TID), or **1950** (50 mg kg^−1^, TID) administered s.c. was initiated at 1 h post-infection and continued for 14 days. Mice were monitored for morbidity and mortality for 37 days. ^#^Significantly different from untreated control (*P* < 0.0001) and the spectinomycin control mice (*P* = 0.0315)

We determined whether treatment with **1950** could protect BALB/c mice from an i.n. infection with 1 x 10^3^ CFU *F. tularensis* strain SchuS4 (10 x LD_50_). Treatment with **1950** (50 or 75 mg kg^−1^) or spectinomycin (50 mg kg^−1^), administered s.c., was initiated at 1 h post-infection and continued for 14 days (BID). Untreated mice (*n* = 10) succumbed to the infection by day 5 and the spectinomycin-treated (*n* = 9, 50 mg kg^−1^, BID) mice succumbed to the infection by day 8 (Fig. 5a). Treatment with **1950** at either dose (50 or 75 mg kg^−1^, BID) resulted in survival of 3/10 mice at day 55 post-infection which was significantly different from the untreated control (*P* < 0.0001) and the spectinomycin control (*P* ≤ 0.0013; Fig. 5a). No bacteria were detected in the lung, spleen, or liver tissue isolated from the mice that survived the infection (data not shown).

Since treatment with **1950** (75 or 50 mg kg^−1^, BID) protected less than half of the mice from *F. tularensis* infection, we also tested whether administration of **1950** (50 mg kg^−1^, TID) could improve survival (Fig. 5b). After infection with 900 CFU *F. tularensis* strain SchuS4, untreated control mice (*n* = 10) succumbed to infection by day 5 and mice that received spectinomycin (*n* = 10; 50 mg kg^−1^, TID) succumbed to infection by day 8. Administration of **1950** (50 mg kg^−1^, TID) resulted in the survival of 4/10 mice, which was significantly increased compared with the untreated control mice (*P* < 0.0001) and the spectinomycin control mice (*P* = 0.0315). Together, these data suggest that amSPC **1950** is a more effective treatment than spectinomycin in the in vivo *F. tularensis* model.

## Discussion

This study demonstrates that amSPCs possess useful antibacterial activity against multiple biothreat pathogens. The modifications to the spectinomycin core rendered these compounds more active than spectinomycin against the Gram-negative pathogens *Brucella* spp., *B. mallei*, and *F. tularensis*. Although there was a slight improvement of activity against the Gram-positive pathogen *B. anthracis* compared with spectinomycin, the MIC values were higher than those which would be preferred for a *B. anthracis* therapeutic. Of the antibiotic combination therapies tested, we determined that most possessed additive or indifferent rather than synergistic effects with compound **1950**, and that the previously noted synergy with macrolides and spectinamides was not analogous to the amSPCs [18]. It is worth noting that streptomycin was antagonistic when combined with compound **1950**, but not with spectinomycin, against *B. anthracis*. This effect was not observed when tested in Gram-negative bacteria, so the issue may be related to the differences between Gram-positive and Gram-negative susceptibilities and uptake of these antibiotics or specific to *B. anthracis* itself. In addition, while streptomycin and spectinomycin bind to the 16S rRNA, they have distinct binding sites, so competition is not expected to be the cause [21].

AmSPC compound **1950** has an improved half-life compared with spectinomycin, and was able to protect mice from *S. pneumoniae* or *N. gonorrhoeae* infections [8, 9]. Here, we investigated the efficacy of amSPC **1950** against two intracellular pathogens, *B. mallei* and *F. tularensis*, which have innate antibacterial resistance [10, 13, 14, 16]. Since amSPCs were able to control the intracellular pathogen *Chlamydia trachomatis* in cell culture, we anticipated that these compounds could gain access to the intracellular *B. mallei* or *F. tularensis* in vivo [8, 9]. While the MIC_90_ was lower for *B. mallei* than for *F. tularensis*, the challenge strains for the two species had the same MIC (12.5 µg ml^−1^). Despite these similar MIC values, compound **1950** had a greater efficacy against *B. mallei* than it did against *F. tularensis*, even when treatment initiation was delayed from 1 to 6 h post-infection. The majority of mice that succumbed to *B. mallei* did so after the treatment with **1950** ended; this corroborates with the increase in bacterial load from day 40 to day 60 post infection in **1950**-treated mice. However, almost half of the mice that succumbed to *F. tularensis* infection did so in the first 14 days post-infection, the time frame for **1950** treatment. These data indicate that compound **1950** reached therapeutic levels in *B. mallei*-infected cells and was able to reduce, but not eliminate, the *B. mallei* infection while the compound was unable to effectively lower *F. tularensis* below lethal quantities. A longer treatment regimen may have improved the survival of *B. mallei*-infected mice, but would likely have no effect on the survival of *F. tularensis*-infected mice.

Compound **1950** had a greater improvement in efficacy in vivo compared with spectinomycin in *F. tularensis*-infected mice than in *B. mallei*-infected mice. The survival of mice administered compound **1950** after *F. tularensis* infection was significantly better than the survival of mice that received spectinomycin, the dose was administered BID or TID. The difference in the MICs of spectinomycin (64 µg ml^−1^) and **1950** (12.5 µg ml^−1^) against the challenge *F. tularensis* strain, SchuS4, explains this difference in survival of mice. The in vitro time-kill assay demonstrated that spectinomycin killed *B. mallei* faster than compound **1950**, despite similar MIC values (12.5 µg ml^−1^ for **1950**, 16 µg ml^−1^ for spectinomycin). However, the survival of mice administered **1950** at 1 h post-infection with *B. mallei* FMH was not significantly different from that of the mice that received spectinomycin, although the **1950**-treated mice had a slightly higher survival percentage (75% compared with 60%). This demonstrates amSPCs may have improved activity compared with spectinomycin against some pathogens, like *F. tularensis*.

Overall, we demonstrated that amSPCs have some useful activity against Gram-negative biothreat pathogens in vitro and in vivo. We further demonstrated the potential for spectinomycin analogs as broad-spectrum antibiotics that function against Gram-negative pathogens of clinical and biodefense importance. Efforts are ongoing to improve the potency of the amSPCs, such as boosting their ability to penetrate Gram-negative pathogens which would lead to a greater level of protection against biothreat pathogens.

## Supplementary information

Supplemental Material
