# Supplementary material for: Aminomethyl spectinomycins: a novel antibacterial chemotype for biothreat pathogens
Source: J Antibiot (Tokyo). 2019 Jun 4;72(9):693–701. doi: 10.1038/s41429-019-0194-8 (PMC6684479; doi:10.1038/s41429-019-0194-8)
Supplement: Supplementary file 1 — Supplemental Material [file 41429_2019_194_MOESM1_ESM.docx]

Table S1. MIC values of *Brucella* spp. to amSPC compounds

|  |  | **Compound (µg/ml)** | | | | |
| --- | --- | --- | --- | --- | --- | --- |
| **Species** | **Strain** | **1950** | **1951** | **2241** | **2324** | **SPT^a^** |
| *Brucella abortus* | 292 | 3.125 | 3.125 | 6.25 | 3.125 | 8 |
| *Brucella abortus* | 870 | 6.25 | 12.5 | 3.125 | 6.25 | 8 |
| *Brucella abortus* | 2308 | 3.125 | 6.25 | 3.125 | 3.125 | 8 |
| *Brucella abortus* | 63/75 | 3.125 | 6.25 | 3.125 | 6.25 | 4 |
| *Brucella abortus* | 86/8/59 | 3.125 | 3.125 | 1.56 | 3.125 | 16 |
| *Brucella abortus* | B3196 | 3.125 | 6.25 | 1.56 | 3.125 | 4 |
| *Brucella abortus* | BT-3 | 3.125 | 6.25 | 3.125 | 3.125 | 4 |
| *Brucella abortus* | BT-4 | 3.125 | 6.25 | 3.125 | 3.125 | 8 |
| *Brucella abortus* | BT-5 | 3.125 | 6.25 | 1.56 | 3.125 | 2 |
| *Brucella abortus* | BT-6 | 3.125 | 6.25 | 3.125 | 3.125 | 4 |
| *Brucella abortus* | C68 | 3.125 | 6.25 | 1.56 | 3.125 | 4 |
| *Brucella abortus* | G8108 | 0.78 | 1.56 | 0.78 | 0.78 | 1 |
| *Brucella abortus* | Tulya | 3.125 | 6.25 | 3.125 | 3.125 | 4 |
| *Brucella melitensis* | 23452 | 0.78 | 1.56 | 0.78 | 0.78 | 2 |
| *Brucella melitensis* | 23456 | 0.78 | 1.56 | 0.78 | 0.78 | 2 |
| *Brucella melitensis* | 16M | 0.39 | 0.78 | 0.78 | 0.78 | 2 |
| *Brucella melitensis* | 63/9 | 0.78 | 1.56 | 0.78 | 0.78 | 2 |
| *Brucella melitensis* | Ether | 0.78 | 3.125 | 1.56 | 1.56 | 2 |
| *Brucella melitensis* | H 26 | 0.78 | 1.56 | 0.78 | 0.78 | 2 |
| *Brucella melitensis* | Rev 1 | 1.56 | 3.125 | 1.56 | 1.56 | 2 |
| *Brucella melitensis* | Strain #101 | 0.78 | 1.56 | 0.78 | 0.78 | 2 |
| *Brucella suis* | 40 | 1.56 | 1.56 | 1.56 | 1.56 | 4 |
| *Brucella suis* | 513 | 12.5 | 3.125 | 1.56 | 6.25 | 32 |
| *Brucella suis* | 686 | 1.56 | 3.125 | 1.56 | 1.56 | 4 |
| *Brucella suis* | 1330 | 0.78 | 0.78 | 0.78 | 0.78 | 4 |
| *Brucella suis* | 23444 | 0.78 | 0.78 | 0.78 | 0.78 | 4 |
| *Brucella suis* | F1183 | 0.78 | 0.78 | 0.78 | 0.78 | 4 |
| *Brucella suis* | Thomsen | 0.39 | 0.39 | 0.39 | 0.39 | 2 |
| *Brucella suis* | Thomsen bv 1152 | 0.39 | 0.39 | 0.39 | ≤ 0.195 | 2 |

^a^ SPT: Spectinomycin

Table S2. MIC values of *B. mallei* strains to amSPC compounds

|  | **Compound (µg/ml)** | | | | |
| --- | --- | --- | --- | --- | --- |
| **Strain** | **1950** | **1951** | **2241** | **2324** | **SPT^a^** |
| NCTC120 | 6.25 | 6.25 | 6.25 | 6.25 | 16 |
| NCTC 10248 | 3.125 | 3.125 | 3.125 | 1.56 | 8 |
| NCTC10229 | 6.25 | 6.25 | 6.25 | 6.25 | 16 |
| NCTC10247 | 6.25 | 6.25 | 12.5 | 6.25 | 8 |
| China 7 | 3.125 | 3.125 | 3.125 | 3.125 | 8 |
| NCTC3709 | 6.25 | 6.25 | 6.25 | 6.25 | 16 |
| 2000031063 | 6.25 | 6.25 | 6.25 | 6.25 | 8 |
| India 86-567-2 | 50 | 50 | 50 | 50 | 16 |
| China 5 | 6.25 | 6.25 | 6.25 | 6.25 | 8 |
| KC237 | 6.25 | 6.25 | 6.25 | 6.25 | 8 |
| KC 1092 | 6.25 | 6.25 | 6.25 | 6.25 | 8 |
| NCTC10260 | 6.25 | 6.25 | 6.25 | 6.25 | 16 |
| FMH | 12.5 | 12.5 | 12.5 | 12.5 | 16 |
| NCTC 3708 | 6.25 | 6.25 | 6.25 | 6.25 | 16 |
| ATCC 10399 | 6.25 | 6.25 | 6.25 | 3.125 | 16 |
| Turkey 1 | 6.25 | 6.25 | 6.25 | 6.25 | 8 |
| Turkey 2 | 6.25 | 6.25 | 6.25 | 3.125 | 16 |
| Turkey 3 | 6.25 | 6.25 | 6.25 | 3.125 | 8 |
| Turkey 4 | 6.25 | 6.25 | 6.25 | 3.125 | 8 |
| Turkey 5 | 6.25 | 6.25 | 6.25 | 3.125 | 16 |
| Turkey 6 | 6.25 | 6.25 | 6.25 | 6.25 | 16 |
| Turkey 7 | 6.25 | 6.25 | 6.25 | 6.25 | 8 |
| Turkey 8 | 6.25 | 6.25 | 6.25 | 3.125 | 16 |
| Turkey 9 | 6.25 | 6.25 | 6.25 | 6.25 | 16 |
| Turkey 10 | 6.25 | 6.25 | 6.25 | 3.125 | 8 |
| 2002721274 | 12.5 | 12.5 | 12.5 | 12.5 | 16 |
| 2002721278 | 3.125 | 6.25 | 6.25 | 3.125 | 8 |
| 2002721279 | 6.25 | 6.25 | 6.25 | 6.25 | 8 |
| 2000031065 | 3.125 | 3.125 | 3.125 | 3.125 | 8 |

^a^ SPT: Spectinomycin

Table S3. MIC values of *F. tularensis* strains to amSPC compounds

|  | **Compound (µg/ml)** | | | | |
| --- | --- | --- | --- | --- | --- |
| **Strain** | **1950** | **1951** | **2241** | **2324** | **SPT**^a^ |
| NIH B-38 | 200 | 200 | 200 | 200 | > 64 |
| LVS var paleartica | 100 | 100 | 100 | 50 | > 64 |
| *F. tularensis* #008 | 12.5 | 12.5 | 12.5 | 6.25 | 64 |
| *F. tularensis* #010 | 12.5 | 6.25 | 12.5 | 6.25 | 32 |
| *F. tularensis* #011 | 6.25 | 6.25 | 12.5 | 6.25 | 32 |
| *F. tularensis* #012 | 6.25 | 12.5 | 25 | 6.25 | 32 |
| Shu4 | 12.5 | 6.25 | 12.5 | 6.25 | 64 |
| JAP (Cincinnati) | 1.56 | 3.125 | 3.125 | 1.56 | 1 |
| VT68 | 12.5 | 12.5 | 12.5 | 12.5 | 32 |
| Larsen NIH-38 | 25 | 25 | 25 | 25 | 64 |
| 425 | 6.25 | 6.25 | 12.5 | 6.25 | 64 |
| Scherm | 6.25 | 6.25 | 6.25 | 3.125 | 32 |
| HOLT | 6.25 | 6.25 | 12.5 | 3.125 | 32 |
| STOLL | 12.5 | 6.25 | 12.5 | 6.25 | 64 |
| OR96-0246 | 25 | 12.5 | 25 | 12.5 | 64 |
| OK00-2732 | 12.5 | 6.25 | 12.5 | 6.25 | 64 |
| OUT02-1927 | 6.25 | 3.125 | 6.25 | 3.125 | 32 |
| MA00-2987 | 12.5 | 6.25 | 12.5 | 3.125 | 64 |
| CA02-0099 | 6.25 | 6.25 | 12.5 | 6.25 | 64 |
| WY96-3418 | 6.25 | 3.125 | 6.25 | 3.125 | 32 |
| KY00-1708 | 12.5 | 6.25 | 12.5 | 6.25 | 64 |
| IN99-1009 | 6.25 | 3.125 | 6.25 | 3.125 | 32 |
| CO01-3027 | 12.5 | 25 | 25 | 6.25 | 64 |
| *F. novicida* #091 | 50 | 25 | 25 | 50 | 64 |
| *F. novicida* #123 | 50 | 25 | 25 | 25 | > 64 |
| *F. novicida* GA99-3549 | 100 | 50 | 50 | 50 | 64 |

^a^ SPT: Spectinomycin

Table S4. MIC values of *B. anthracis* strains to amSPC compounds

|  | **Compound (µg/ml)** | | | | |
| --- | --- | --- | --- | --- | --- |
| **Strain** | **1950** | **1951** | **2241** | **2324** | **SPT**^a^ |
| V770-NP1-R | 25 | 25 | 12.5 | 25 | 32 |
| New Hampshire | 50 | 25 | 12.5 | 25 | 64 |
| Vollum | 50 | 50 | 12.5 | 25 | 64 |
| Ames | 50 | 25 | 12.5 | 25 | 64 |
| Sterne | 50 | 50 | 12.5 | 25 | 64 |
| *B. anthracis* #035 | 25 | 25 | 12.5 | 25 | 64 |
| Smith 1013 | 25 | 12.5 | 12.5 | 12.5 | 32 |
| DeltaANR | 50 | 50 | 12.5 | 50 | 64 |
| *B. anthracis* #110 | 25 | 25 | 12.5 | 50 | 64 |
| Vollum 1B | 50 | 25 | 12.5 | 25 | 64 |
| Zimbabwe 89 | 50 | 50 | 12.5 | 25 | 64 |
| SK-102 (Pakistan) | 50 | 50 | 12.5 | 25 | 64 |
| LA-1 Etosha National Park | 25 | 25 | 12.5 | 25 | 64 |
| BA0052 | 25 | 25 | 12.5 | 25 | 32 |
| G-28 | 25 | 12.5 | 12.5 | 25 | 64 |
| *B. anthracis* #140 | 25 | 25 | 12.5 | 25 | 64 |
| German LVS | 25 | 12.5 | 12.5 | 25 | 32 |
| Canadian bison | 25 | 12.5 | 6.25 | 12.5 | 32 |
| Pasteur | 25 | 12.5 | 6.25 | 12.5 | 32 |
| South Africa (BA1035) | 25 | 25 | 12.5 | 25 | 64 |
| EB-1 | 25 | 12.5 | 12.5 | 12.5 | 64 |
| B7T5 | 25 | 25 | 12.5 | 25 | 64 |
| RA3 | 25 | 25 | 25 | 25 | 32 |
| Ba1015 | 25 | 25 | 12.5 | 25 | 64 |
| Ohio ACB | 50 | 25 | 12.5 | 25 | 64 |
| Turkey #32 | 50 | 25 | 12.5 | 25 | 64 |
| A0435 | 100 | 25 | 25 | 50 | 64 |
| 2000031021 | 12.5 | 6.25 | 3.125 | 12.5 | 16 |
| 2002013094 | 25 | 12.5 | 6.25 | 12.5 | 32 |
| 2000031052 | 25 | 25 | 12.5 | 25 | 32 |

^a^ SPT: Spectinomycin

Table S5. MIC values of *B. pseudomallei* strains to amSPC compounds

|  | **Compound (µg/ml)** | | | | |
| --- | --- | --- | --- | --- | --- |
| **Strain** | **1950** | **1951** | **2241** | **2324** | **SPT**^a^ |
| K96243 | > 200 | > 200 | > 200 | > 200 | > 64 |
| Pasteur 52237 | > 200 | > 200 | > 200 | > 200 | > 64 |
| 7894 | > 200 | > 200 | > 200 | > 200 | > 64 |
| MSHR305 | > 200 | > 200 | > 200 | > 200 | > 64 |
| MSHR668 | > 200 | > 200 | > 200 | > 200 | > 64 |
| NAU20B16 | > 200 | > 200 | > 200 | > 200 | > 64 |
| NAU35A03 | > 200 | > 200 | > 200 | > 200 | > 64 |
| 1026b | > 200 | > 200 | > 200 | > 200 | > 64 |
| MSHR5855 | > 200 | > 200 | > 200 | > 200 | > 64 |
| MSHR5848 | > 200 | > 200 | > 200 | > 200 | > 64 |

^a^ SPT: Spectinomycin
